# Metal artifact correction strategies in MRI-based attenuation correction in PET/MRI

**DOI:** 10.1259/bjro.20190033

**Published:** 2019-11-14

**Authors:** Georg Schramm, Claes Nøhr Ladefoged

**Affiliations:** 1 Department of Imaging and Pathology, Division of Nuclear Medicine, KU/UZ Leuven, Leuven, Belgium; 2 Department of Clinical Physiology, Nuclear Medicine and PET, Rigshospitalet, Copenhagen, Denmark

## Abstract

In hybrid positron emission tomography (PET) and MRI systems, attenuation correction for PET image reconstruction is commonly based on processing of dedicated MR images. The image quality of the latter is strongly affected by metallic objects inside the body, such as *e.g*. dental implants, endoprostheses, or surgical clips which all lead to substantial artifacts that propagate into MRI-based attenuation images. In this work, we review publications about metal artifact correction strategies in MRI-based attenuation correction in PET/MRI. Moreover, we also give an overview about publications investigating the impact of MRI-based attenuation correction metal artifacts on the reconstructed PET image quality and quantification.

## Introduction

To reconstruct positron emission tomography (PET) images with correct regional contrast and absolute quantification, correction for photon attenuation is essential. Commonly, attenuation correction (AC) is performed by using a 511 keV transmission scan in standalone PET systems or by multilinear rescaling of a CT image in hybrid PET/CT systems. In hybrid PET/MRI systems, where no direct information about photon attenuation is available, AC is currently based on processing of MR images (MRI-based attenuation correction, MRAC). On top of the classical MRAC problems such as the difficulty to reliably represent cortical bone in MR images or the limited transaxial field of view of MRI systems, the presence of metallic implants poses a major challenge for MR imaging and thus for MRAC. In this review about metal artifact correction strategies in MRAC for PET/MRI, we first give an introduction to metal artifacts in MR imaging and briefly summarize the state of the art for MRAC in clinical systems and research in the “Introduction” section. The following section describes the prevalence and impact of MRAC metal artifacts on the reconstructed PET image quality and quantification. In the “Correction strategies for MRAC metal artifacts” section, we review published correction strategies for MRAC metal artifacts before closing with a discussion and outlook. Since MRAC processing and its impact on the reconstructed PET images is substantially different for brain compared to whole-body PET/MRI acquisitions, we discuss them separately.

### Metal artifacts in MR imaging

Metallic objects inside the body pose a severe challenge for MR imaging. Despite the fact that many metal implants are MRI safe, they strongly interfere with the MRI signal acquisition and thus affect the MR image quality that can be reconstructed. In the following, we briefly summarize the most common MR image artifacts that are caused by metal implants such as, *e.g*. dental fillings, surgical clips, screws or endoprostheses. For a more detailed problem description, we refer to the review of Hargreaves et al.^1^

First, due to the lack of hydrogen nuclei in metal implants, no transverse magnetization, which is used as the imaging signal in MRI, is created. Consequently, the metal implant itself appears without signal (black) in the reconstructed MR image. Second, the presence of metal can cause strong variations in the static magnetic B_0_ field due to susceptibility changes between the metal implant and the surrounding tissue. Those B_0_ field variations depend on size, shape, type, and orientation of the metal implant and lead to local variations in the Larmor (resonance) frequency causing dephasing and signal loss. In the vicinity of metal objects, the spatial variations in the Larmor frequency can be so strong that magnitizations within a single voxel precess at different frequencies leading to complete dephasing and signal loss. As a consequence, regions around metal implants also often appear without signal in the reconstructed MR images. Commonly, the superposition of the first two effects results in a region without any MRI signal that is bigger than the metal implant itself (signal void or halo effect). The underlying dephasing of the magnetization can be reduced by using spin echo sequences or gradient echo sequences with very short echo times.^1^

**Figure 1.**
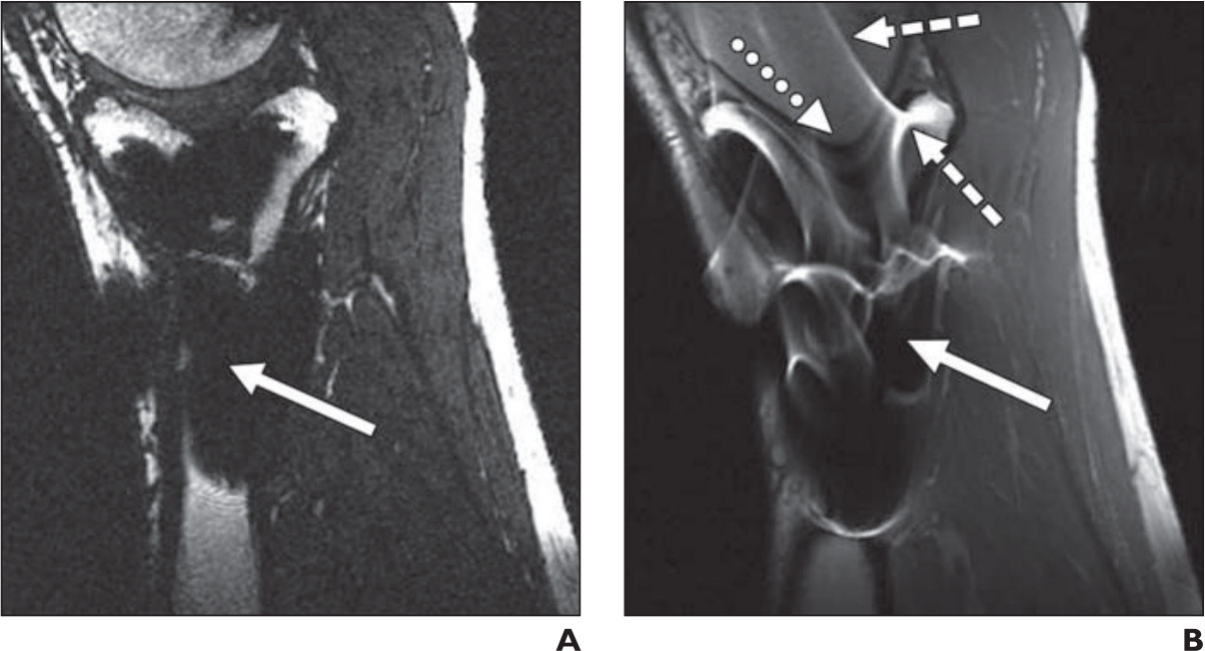
Examples of artifacts due to presence of stainless steel screws in healthy 37-year-old male. A and B, In gradient-echo image with *±*62.5 kHz receive bandwidth (A) and spin-echo image with *±*16 kHz receive bandwidth (B), solid arrows show signal loss that can be due to dephasing or from signal being shifted away from region. Dotted arrow in B shows geometric distortion of femoral condyle, and dashed arrows show signal pile-up, which can be combination of in-plane and through-slice displacement of signal from multiple locations to one location. Reprinted from “Metal-induced artifacts in MRI”, B. Hargreaves et al., *the American Journal of Roentgenology* 197/3, © 2011 American Roentgen Ray Society.

The spatial variations in the resonance frequency can lead to displacement of signal during excitation and readout which can shift signal away from regions (signal loss) but also to signal accumulation in other regions (signal pile-up). Another consequence of the resonance frequency variations can be geometric distortions and failure of water fat separation. Dixon techniques that are often used in MRAC can detect low frequent variations in the B_0_ field but also fail to separate water and fat close to metal implants.^1^ A typical example showing different MRI artifacts induced by a metal screw is shown in Figure 1.

### MRI-based attenuation correction in clinical PET/MRI systems and research

Since the introduction of PET/MRI for clinical use almost a decade ago, there has been a dedicated focus on improving MRAC.^2–7^ The majority of the focus has been on the brain, which can be attributed to two main factors. First, the brain is, due to its rigid nature, an easier task for registration methods compared to the rest of the body.^8^ Second, the initial MRAC problem of ignoring higher attenuation values of bone has more impact in the brain compared to the rest of the body. This is because the entire organ of interest (the brain) is completely enclosed by bone (the skull) making the exclusion of bone result in major PET bias in PET/MR imaging both close to and distant from the skull.^9^ The main motivation for the advances in brain MRAC has been the acknowledgment of the importance of correctly accounting for bone during MRAC.

Today, MRAC is generally considered a solved topic for adult brains with normal anatomy.^7^ However, an often neglected problem is MRAC in the presence of metal objects. Whereas a metal implant tends to produce streak artifacts in CT images,^10^ it affects the MR images by inducing a susceptibility void with a reduced or complete lack of MRI signal.^11^ As mentioned before, the size of the artifact area depends on the type of metal, and the MRI sequence used,^12^ but is often greater than the extent of the metal itself.^12^ The distortion can in some cases make the image anatomically unrecognizable.^12^ The resulting local errors in the MR-based attenuation image introduced by neglecting tissue and metal in the affected area in the MR image processing translate into a bias in the reconstructed PET images.^6,13–18^ Patients referred for a PET/MRI examination are requested to remove all metal where possible,^19^ but some metal cannot easily be removed. The main sources of the remaining metal-induced artifacts in brain PET/MRI are dental implants and surgical materials and devices.

MRAC in the body is in general more challenging compared to MRAC in the brain. This is because, first, most organs within the body move non-rigidly. Examples for non-rigid organ motion or deformations are respiratory or cardiac motion, flexible positioning of the arms, or non-rigid compression of the body caused by whole-body MRI coils. Second, the anatomic variability in the body is much bigger than within the brain which complicates the use of atlas-based techniques in whole-body MRAC for a wide range of patients.

That is why, all vendors started to use segmentation-based techniques to generate 3-class (Philips: air, lung, soft-tissue segmentation of *T*_1_ weighted MR image^20,21^) or 4-class (Siemens, GE: air, lung, fatty tissue soft-tissue segmentation of Dixon type MR images^22^) attenuation images with fixed attenuation values assigned to the different tissue classes in whole-body PET/MRI. Note, that the missing bone problem is also present in whole-body MRAC. However, the introduced bias is much more localized to structures within or close to bone^23,24^ and thus in general less severe than in brain applications. Recently, Paulus et al.^25^ evaluated a model-based bone segmentation algorithm of Dixon images using a set of prealigned MR image and bone mask pairs for all major bones in the body. This technique is now implemented in the latest version of the Siemens mMR.

**Figure 2.**
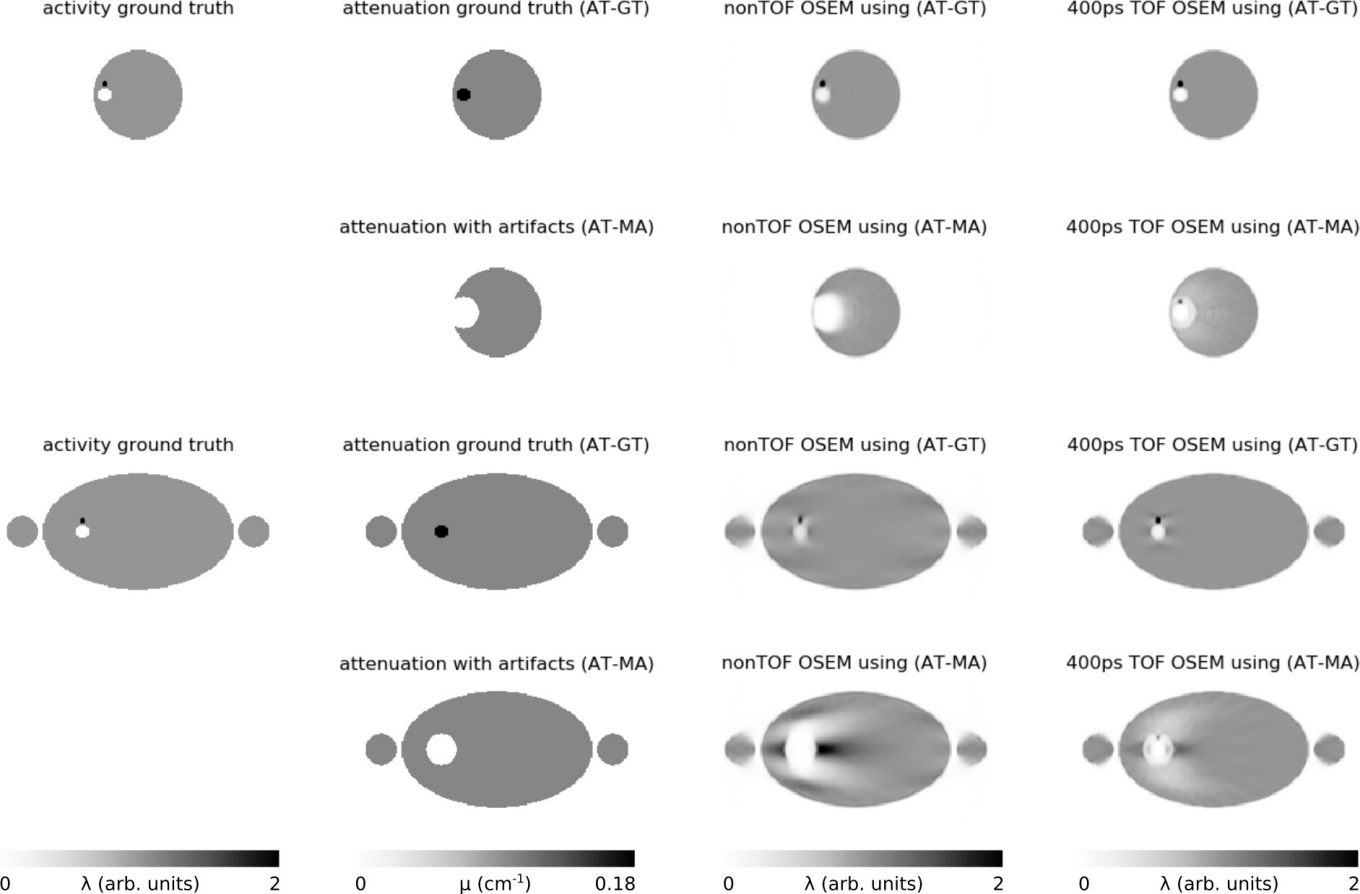
3D Noise-free simulation of the impact of metal artifacts in the attenuation image in non-TOF and 400 ps TOF OSEM reconstructions (only transaxial slices are shown). The top two rows show the case of a signal void caused by a dental implant in the head. The bottom two rows show the case of a signal void caused by a hip implant in the body. The first column shows the activity ground truth used to simulate the emission data. The second column shows the ground truth attenuation image and the attenuation image with a simulated metal artifact that were used for PET image reconstruction. The last two columns show non-TOF and 400 ps TOF OSEM PET reconstructions (3 iterations with 28 subsets, 2.78 × 2.78 × 2.78 mm^3^ voxelsize, 600 mm transaxial FOV, 4.5 mm post-smoothing) using both attenuation images. The PET scanner geometry used in this simulation was the one of the GE SIGNA PET/MRI. The used linear attenuation coefficients were 0.1 cm*^−^* ^1^ for soft tissue and 0.18 cm*^−^* ^1^ for the implant.

In general, all MRI segmentation-based methods for the generation of PET attenuation images face problems in regions with no MRI signal or contrast. Examples for those regions are:

- Regions outside the MRI field of view, which is smaller than the field of view of whole-body PET scanners (ca 45 *vs* 60cm), lead to truncation artifacts in MRI segmentation-based attenuation images. Completion of those regions with the soft tissue attenuation coefficients is commonly done by using information from PET images reconstructed without or with imperfect AC.^26,27^
- Cortical bone regions with extremely short T2 times. The latter and the fact that trabecular bone can show a variety of signals in different MRI sequences due to its fat content, currently impedes reliable segmentation of bones in T1 or Dixon-based MRI sequences without prior information
- Regions inside the lung containing lung tissue with low MRI signal. Segmentation of the lungs is usually performed by using anatomical prior knowledge (regions with low MRI signal above a certain volume in the chest). Segmentation of the lungs is in general quite stable unless the low signal regions of the lungs are connected to other low signal regions such as the background, *e.g*. due to metal artifacts caused by sternal cerglages.
- Regions inside and around metal implants such as hip, knee, or shoulder endoprotheses, spinal disk implants, sternal cerclages, surgical clips, or injection ports used for chemotherapy. As discussed in the “Metal artifacts in MR imaging” section, metallic implants produce susceptibility artifacts in MRI scans that lead to signal voids in and around the implant. Usually, those artifact regions are assigned with the air attenuation coefficient due to their low MRI signal. This in turn produces regions in the attenuation images where the attenuation is locally underestimated.^28,29^ The magnitude and size of the introduced bias (usually underestimation) in the reconstructed attenuation corrected PET images depends on the size of the artifact and the time of flight (TOF) resolution that is available in the PET reconstruction. An example of a typical metal implant induced MRAC artifact is shown in Figure 2in reference.^30^

In the following section, we briefly discuss the impact of metal-implant induced MRAC artifacts on the reconstructed PET images.

## The prevalence and impact of metal-implant induced MRAC artifacts on the reconstructed PET image quality and quantification

### Theoretical considerations

In clinical practice and research, PET image reconstruction is commonly performed using the iterative early-stopped (ordered subset) maximum likelihood expectation maximization algorithm (OSEM). In OSEM, the update of the activity concentration ***λ*** in a voxel *j* is given by

(1) $$\lambda_{j}^{+}=\frac{\lambda_{j}}{\sum {}_{i}\sum {}_{t}c_{itj}}\sum_{i}\sum_{t}c_{itj}\frac{y_{it}}{{\bar{y}}_{it}(\lambda )}$$

where *y_it_* denotes the measured coincidence data in TOF bin *t* on line of response (LOR) *i* and *c_itj_* denotes the system matrix. $\overset{-}{y_{it}}(\lambda )$ is the estimated coincidence data based on the current image estimate given by

(2) $$\bar{y}{}_{it}(\lambda )=\sum_{j}c_{itj}\lambda_{j}+s_{it}$$

where *s_it_* are additive contaminations such as scattered and random coincidences. The system matrix elements *c_itj_* are commonly factorized into

(3) $$c_{itj}=n_{i}\;a_{i}\;(\mu )\;p_{ij}\;g_{itj}$$

where *p_ij_* are geometric forward projection weights, *g_itj_* is the TOF kernel, *n_i_* is a normalization (sensitivity) factor, and the AC factor *a_i_* for LOR *i* given by

(4) $$ln\;a_{i}\;(\mu )=-\sum_{j}l_{ij}\mu_{j}$$

where *µ_j_* is the 511-keV linear photon attenuation coefficient (LAC) in voxel *j*. Note that local errors in the attenuation image ***µ*** lead to errors in part of the AC factors and hence to inconsistencies in part of the system matrix. As an example, local underestimations in the attenuation image, which are common for metal-implant based MRAC artifacts, lead to overestimations of part of the attenuation factors and system matrix elements.

The specific impact of metal-based MRAC inconsistencies in the system matrix on the reconstructed activity image ***λ*** is in general hard to predict, since it depends on different factors such as the distribution of activity and attenuation and the available TOF resolution. Conti et al.^31^ already noticed in 2011 that TOF PET reconstruction is more robust in the presence of inconsistent data. In this work, the authors concluded that TOF PET reconstruction “is less sensitive to mismatched attenuation correction, erroneous normalization and poorly estimated scatter correction. The spatial weighting derived from the TOF information competes, when conflicting, with other spatial weighting (normalization and attenuation), and during reconstruction partially corrects the accumulation of activity in parts of the image that are inconsistent with the data timing information. Such robustness is directly dependent from the time resolution of the TOF PET scanner, so we expect new generations of PET scanners, with improved time resolution, to be less and less sensitive to poor quality normalization, scatter and attenuation corrections. Moreover, TOF reconstruction can be beneficial in multimodalities, such as PET/MRI, where a direct attenuation measurement is not available and attenuation correction can only be approximated.”

To visualize the potential impact of typical MRAC metal artifacts as a function of the available TOF resolution, we performed reconstructions of noise-free simulated PET emission data using the true attenuation image (the one that was used for data generation) and an attenuation image containing a typical metal MRAC artifact, namely a misclassification of the metal implant and its surrounding as air. The PET scanner geometry used in this three-dimensional simulation was the one of the GE SIGNA PET/MRI. Data of a head (dental implant artifact) and pelvic bed position (unilateral hip endoprosthesis) were simulated and reconstructed without TOF information and with a TOF resolution of 400 ps using 3 OSEM iterations with 28 subsets and 4.5 mm gaussian post-smoothing. As expected, the reconstructions shown in Figure 2 demonstrate that locally the simulated metal MRAC artifacts lead to a substantial underestimation of the reconstructed activity concentration in both the non-TOF and 400 ps TOF reconstruction. In line with the results shown in Conti et al.,^31^ the magnitude and the extent of the local underestimations is smaller in the 400 ps TOF reconstruction. The simulated lesion close to the implant is obscured in the non-TOF reconstruction, but remains visible in the TOF reconstruction*—*although with a substantial negative bias. Interestingly, the local underestimation of the attenuation around the implant in the pelvis also leads to local positive bias in the horizontal direction outside the artifact region. This bias is again more pronounced in the non-TOF reconstruction.

### Brain PET/MRI

**Figure 3.**
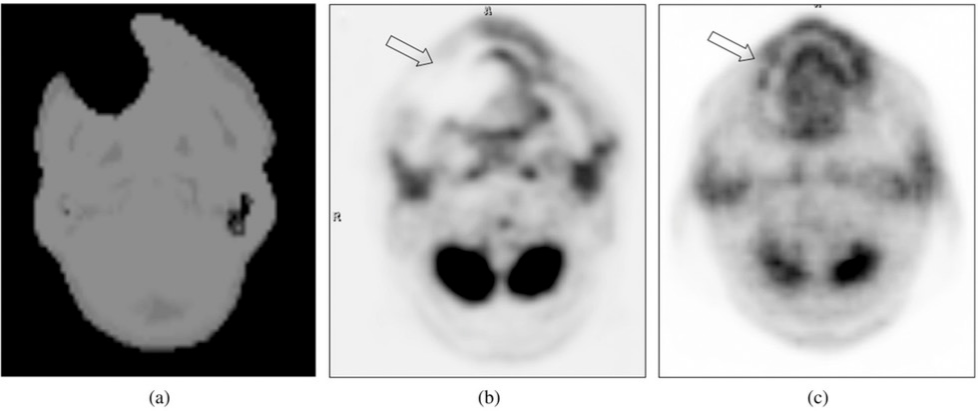
The MR-based attenuation image showing a typical artifact caused by a dental implant. (a) Shows a large region of missing data and tissue misclassification as air around the dental implants, resulting in an artificially decreased ^18^F-FDG uptake (arrow) in the corresponding MRAC PET (b) relative to the CT-based attenuation-corrected PET (c). Figure adapted from Buchbender et al. “Positron emission tomography (PET) attenuation correction artefacts in PET/CT and PET/MRI”, *Brit J Radiol* 2013, 27:1–9^13^ and reused with permission from the publisher, BIR.

Dental implants have a high prevalence,^32^ since they can be present in patients across all clinical indications and especially in elderly patients. Ladefoged et al.^32^ found clear visual artifacts in 44% of 339 investigated patients with Dixon-based MRAC in the Siemens mMR. An example of a dental artifact in a Dixon-based MRAC map and the bias introduced in the PET image is illustrated in Figure 3. Buchbender et al.^13^ investigated the likelihood of MRAC artifacts based on the presence of MRI artifacts. The authors found that out of 20 dental implants, 12 of them presented with visual artifacts on the MR images, and all but one of these impacted the MRAC images to a degree that resulted in decreased PET uptake. An example of the introduced bias was illustrated by Gunzinger et al.,^33^ who simulated dental artifacts of varying sizes inserted into the attenuation image in or near a carcinoma in the right tonsil, and found maximum SUV differences in the range of 8–33% from metal implants of 1–5 cm diameter for the GE trimodality PET/MRI system with 544 ps TOF resolution. Metal objects such as surgical materials and devices can be present especially when examining brain tumor patients post surgery.^34^

The impact on MRAC of a metal-induced artifact in an MR image depends on the type of MRAC utilized, which can in large be split into three categories: segmentation-based, atlas-based and emission-based. Since the methods utilize and depend on the MR images differently, the influence of artifacts therefore also affect the methods in each category differently.

In the segmentation-based methods, where the image is separated into tissue classes based on the relative signal intensity, a signal void will most often be assigned to the air class,^13^ whereas high intensity areas at the border of the signal void^12^ will be assigned to the bone class when segmentation is based on UTE^14^ or ZTE MRAC sequences.^16^ The attenuation of the metal itself is not accounted for using the segmentation-based methods. A metal artifact-induced signal void therefore typically underestimates the PET uptake due to both the metal and soft tissue areas being set to air in the attenuation map,^13^ that can impact the clinical setting when evaluating pathology close to the metal implant.^14^

In the atlas-based methods, the MR image is used for registration and database matching. In the registration step, the MR image is non-linearly registered to one or more of the database-MRIs.^35–38^ Larger artifacts with complete signal voids diminish the accuracy of the registration locally.^36^ The corresponding CTs are typically combined into a pseudo-CT by averaging over a patch. The CTs are selected based on the corresponding MRIs similarity to the subject’s MRI. When the subject MRI is lacking signal, finding the best matching MRIs in the database is challenging.^36^ Confidently obtaining a matching MRI patch that in fact represents the same area is near impossible.

In the emission-based methods, where the MR image is only used as an anatomical prior, metal artifacts influences the predicted MR-based attenuation image to a lesser degree.^39^

### Whole-body PET/MRI

The prevalence of metal artifacts in whole-body MRAC strongly depends on the clinical indication, previous treatments (*e.g.* surgeries, chemotherapy) and age (prevalence of hip, knee and spine implants) of the examined patients. Brendle et al.^40^ found 44 metal artifacts in 100 patients with subsequent PET/CT and PET/MRI (Siemens mMR) examination (mostly oncological indications, mean age 57 years). The metal artifacts in the attenuation image quantitatively affected 21% of all PET-avid lesions (38 out of 184). However, they did not affect the diagnosis. Correction (inpainting with soft tissue LAC) of the metal artifacts increased the SUV in from 1.6 *±* 1.1 to 6.3 *±* 2.2 in five lesions. In a prospective PET/MRI study by Seith et al.,^41^ 18 out of 200 (9%) consecutively enrolled oncological patients had to be excluded due to significant movement or large metal artifacts. Schramm et al.^42^ reported that the attenuation images of 19 out of 316 consecutively enrolled whole-body patients (6%) examined with a Philips Ingenuity TOF PET/MRI were affected by metal artifacts. Correction of the metal artifacts in the attenuation image (automatic inpainting with soft tissue LAC) lead to increase of up to 59% in the reconstructed SUV.

**Figure 4.**
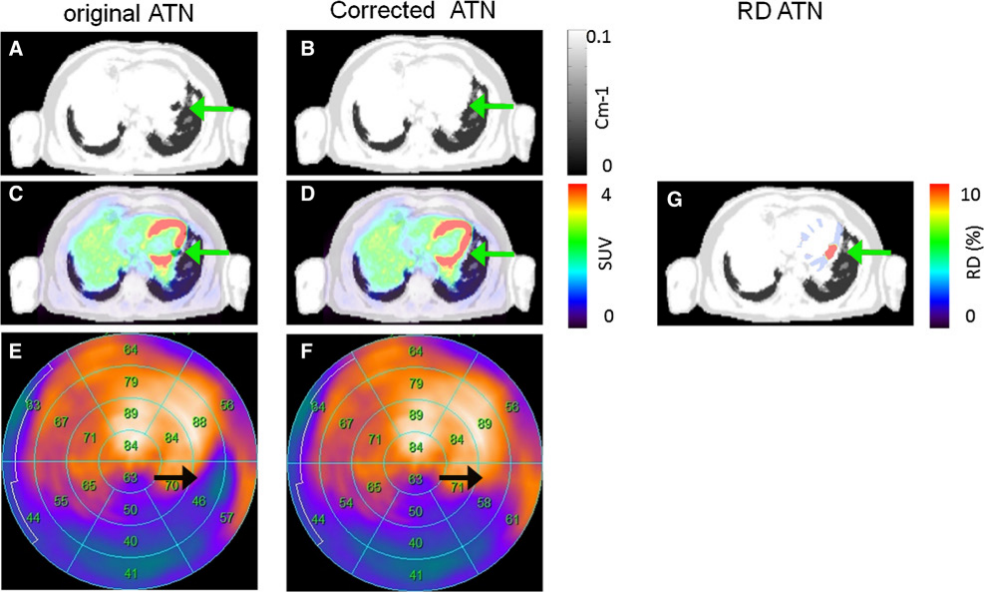
Patient 13 with susceptibility artifact in the inferior wall caused by a stent and corresponding FDG PET reconstructions. Susceptibility artifact in the left circumflex artery was observed in the original AC map (A, arrow). Correction of the susceptibility artifact (B) changed the interpretation from reduced metabolism to normal metabolism (C*–*F, arrows). The susceptibility artifact accounted for relative differences of more than 10% in the affected region (G). This research was originally published in J Nucl Cardiol. Lassen et al. “Assessment of attenuation correction for myocardial PET imaging using combined PET/MRI” *J Nucl Cardiol*. 2017.^48^ This figure is reprinted without modifications and is licensed under the Creative Commons Attribution 4.0 International License.

Davison et al.^43^ simulated the impact of metal artifacts in MRI-based attenuation images by introducing artificial regions with air attenuation of different sizes at different locations in seven patients examined with the GE SIGNA TOF PET/MRI. The resulting underestimation in the reconstructed activity in the artifact locations was up to 97% (lumbar spine) in non-TOF reconstructions and up to and up to 63% (thoracic spine) when using TOF in the reconstruction. The fact that the availability of TOF during PET reconstruction reduces bias introduced by metal artifacts in the attenuation image was also demonstrated by Mehranian et al.^44^ in a subject with a hip endoprotheses acquired with the Philips Ingenuity TOF PET/MRI. A study by ter Vort et al.^30^ analyzed 65 patients who underwent a scan on the GE SIGNA TOF PET/MRI and found that 4 patients (6%) had large and 8 patients (12%) small metal implant related artifacts in the attenuation image. Moreover, 27 patients (41%) showed artifacts due to dental implants. Reading by two certified radiologists/nuclear medicine physicians revealed that the impact on the perceived image quality of the reconstructed PET images was less severe in TOF *vs* non-TOF PET reconstructions. In a patient with thoracolumbar spinal fusion, a bone metastasis was obscured in the non-TOF reconstruction, but could still be identified in the TOF reconstruction. Svirydenka et al.^45^ studied the detectability of lesions close to simulated signal voids in non-TOF and TOF reconstructions in 27 patients with 51 lesions adjacent to the signal voids. In nine and one lesions, the detectability changed for non-TOF and TOF reconstructions, respectively. Two lesions were no longer visible in the non-TOF reconstructions. In all lesions, the SUV underestimation was decreased in TOF compared to non-TOF reconstructions. For example, in a lesion close to a simulated hip implant, the bias decreased from ca. 82% in the non-TOF reconstruction to ca. 42% in the TOF reconstruction. Attenberger et al.^46^ showed two clinical examples of MRAC metal implant artifacts caused by surgical clips and a port used for chemotherapy and concluded “Since there are currently no sufficient technical solutions for the reduction of metal artifacts in PET/MRI, the best practice at this point is to simply perform PET-CT imaging if pathology is suspected adjacent to metallic implants.” Delso et al.^47^ also concluded that at this moment (2015), there is no standard way to compensate for MRAC metal artifacts. In their PET/MRI study of myocardial viability, Lassen et al.^48^ found that the attenuation images in 10 out of 20 cardiac patients (50%) suffered from susceptibility artifacts caused by sternotomy or other metallic objects such as stents. An example where an MRAC metal artifact changed the interpretation of a polar map in cardiological PET/MRI is shown in Figure 4.

Olin et al.^49^ investigated the reproducibility of MRI-based thoracic attenuation images in the Siemens mMR. 1 of the 11 analyzed patients showed metal artifacts in each of the two generated attenuation images of all four PET/MRI examinations. The reproducibility of the SUV_max_ in the second of the two lesions in that patient was not better than ca. 18%. Kuttner et al.^50^ analyzed the reproducibility of lesion quantification in 25 lung cancer patients using the Siemens mMR. Three cases were affected by susceptibility artifacts that led to an underestimation of the reconstructed activity of up to 20% and “large inconsistencies between test-retest scans.”

## Correction strategies for MRAC metal artifacts

### Brain MRAC

Despite that most of the focus in PET/MRI AC research has been on the brain, the amount of metal artifact correction methods are limited. The methods are, roughly put, based on dedicated sequences, an (un)intended feature of the MRAC method, or a post-processing of the traditional MRAC images. The advances within each area are described in the following.

#### Dedicated MRI sequences

Metal artifacts in MR images appear differently depending on the acquisition sequence utilized*—*they are most severe in gradient echo sequences, and most reduced in spin echo sequences with short TE.^51,52^ Specialized sequences allow for imaging near smaller metal objects, where examples hereof include MAVRIC^53^ and SEMAC,^54^ or hybrids MAVRIC-SEMAC^55^ and UTE-MAVRIC.^56^

In a prospective study, Gunzinger et al.^33^ examined the effect of using MAVRIC to reduce dental artifacts. The authors acquired PET/CT and MRI scans for 25 patients, including standard MAVRIC and an adapted version optimized for speed. The artifacts present in the images were manually measured using the largest diameter in the axial direction and assuming ellipsoidal shape. Using the MAVRIC sequences, it was found that the size was significantly reduced (−75%) compared to a standard MRAC LAVA-Flex sequence.

**Figure 5.**
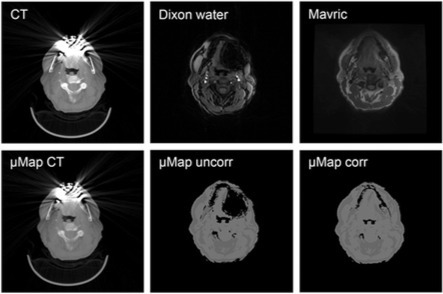
(Top) Typical examples of images obtained from low-dose CT, water data set of Dixon sequence, and MAVRIC sequence are shown. (Bottom) Corresponding attenuation maps generated from CT, Dixon sequence alone, and new correction algorithm are shown. This research was originally published in JNM. Burger, I. A. et al. “Hybrid PET/MRI imaging: an algorithm to reduce metal artifacts from dental implants in Dixon-based attenuation map generation using a multi acquisition variable resonance image combination sequence”. *J Nucl Med*. 2015;56:93–97. ^©^ SNMMI.^57^

In a retrospective study, Burger et al.^57^ extended this, by similarly utilizing a diagnostic MAVRIC sequence to correct the Dixon-based attenuation maps in areas with dental implants. The method relies on the tissue intensity of the MAVRIC image in artifact areas where the Dixon in- and opposed-phase MR images have a lack of signal. By superimposing either fatty or soft tissue values in the artifact area, based on the MAVRIC image intensity, the authors obtained a corrected attenuation map. An illustration of the performance can be seen in Figure 5. In eight patients, the artifacts in the MRAC maps were reduced on average from 698 to 203 mm^2^ when using the semiautomatic correction method.

The added accuracy of the specialized sequences also comes at the cost of increased acquisition time which is usually too high for pure MRAC purposes*—*the acquisition time of the MAVRIC sequence is between 3.5 and 6 min, depending on the version, compared to 14 s for a LAVA-Flex MRAC sequence.^33^ It could, however, be of interest in specialized occasions, *e.g*. when examining patients with tumors in the oropharynx.^33^

#### Part of the attenuation method

Metal artifact correction is in some MRAC methods already a feature by the very nature of the method type, either coincidentally or by design. The following highlights how novel MRAC methods handle metal artifacts.

##### Joint estimation of emission and attenuation

When AC is based on emission data, the influence of the MR image is less or non-existing compared to a segmentation-based method, making such methods robust to metal artifacts.^58^ Mehranian et al.^39^ utilized an MRI prior for brain MRAC using maximum likelihood activity and attenuation (MLAA) reconstruction, and found that the emission-based method had a high potential to differentiate metallic implants from soft tissues when using TOF scanners, and even to recover the high valued linear attenuation coefficients (LACs) of dental implants. In general, joint estimation of activity and attenuation methods are not yet accepted as a clinically feasible MRAC technique, mainly due to the fact that they require sufficient TOF resolution, due to the limited accuracy compared to segmentation- or atlas-based methods,^7,39^ and due to the absence of reliable implementations into clinical PET/MRI scanners. With it being an active field of research, the former limitations are likely to be overcome. Recently, a publication by Razaei et al.^59^ showed improved performance of a TOF-based MLAA MRAC method in brain PET/MRI, with accuracy similar to a state-of-the-art ZTE-based attenuation method for data from the GE SIGNA TOF PET/MRI. The authors demonstrated the ability of their method to recover most of the jaw, including bone and metal, in a patient with metal implant-induced artifact in the dental region, despite using an MRI-based intensity prior as input (Figure 4 in Rezaei^59^).

##### Atlas-based

In the case of smaller metal artifacts occurring at a common location, as in dental artifacts, the atlas-based MRAC methods could be a viable solution. By matching to the database MRIs, the atlas-based methods can select cases with similar artifacts. The use of pattern or patch matching might even introduce metal into the attenuation map in the cases of moderate dental artifacts.^39,60^ Hofmann et al.^61^ showed how an atlas-based method reduced the effect of dental artifacts from 40% PET underestimation to <10% compared to conventional segmentation-based MRAC. Atlas-based methods are, due to this relative insensitivity to metal, superior to segmentation-based methods in the case of smaller dental artifacts.^4^

In the case of larger artifacts, completely removing the MRI signal, or artifacts from surgical materials occurring at uncommon locations, atlas-based methods are challenged by the lack of training data. Especially atlas-based^35,36,38^ or pattern-recognition methods^37^ that synthesize the CT image within local patches that might be completely corrupted are challenged. In such cases, a template-based method, which is a subtype of the atlas-based methods only utilizing a single MRI-CT pair, might provide more accurate and anatomically correct results for the missing artifact region, as it retains the spatial relative information.^62,63^

It is worth noting that the atlas-based methods are further challenged by a lack of a true gold-standard, since CT images also suffer from metal implants resulting, *e.g*. in streak artifacts. Moreover, the rescaling of CT Hounsfield units of metals to 511 keV attenuation coefficients is non-trivial.

##### Deep learning-based

Recently, several methods for brain MRAC have been proposed utilizing the advances within deep learning and convolutional neural networks.^64–68^ These networks excel in noise reduction, and could therefore contain an untapped potential for metal artifact correction, as has been explored towards CTAC metal artifact reduction.^69,70^ Ladefoged et al.^68^ showed that a deep learning-based MRAC method was able to achieve a performance comparable to CTAC in pediatric patients with metal clip implanted into the skull causing both artifacts and abnormal anatomy in the used MR images. Recently, Hwang et al.^71^ trained a deep learning model with the activity and transmission outputs of a TOF-based MLAA algorithm to improve the accuracy of the attenuation map, and achieved relative PET errors comparable to other state-of-the-art MRAC methods.^7^ Similar to the emission-based methods, this method works independent of the MR images, making it insusceptible to metal artifacts. It remains to be shown whether deep learning-based methods can play a role in correcting larger metal artifacts.

### Post-processing of the attenuation maps

Post-processing of the attenuation map is the only remaining strategy to correct for metal artifacts when neither specialized sequences is an option or the chosen MRAC method manages to overcome the artifact. Ladefoged et al.^72^ proposed a twofold method to correct dental artifacts by first delineating the outer contour of artifacts breaching the anatomical surface using statistical models of possible dental shapes, and then classifying internal air cavities based on their position relative to anatomical landmarks. This type of method is limited to inserting soft tissue in the signal voids, failing to compensate for the metal implant itself. When comparing the PET signal after filling the artifact region with soft tissue, which is still an underestimation of the true density, the mean PET signal increased by 48% in the affected region, and up to 7% in the cerebellum.^32^ Here, it was concluded that the artifact region could potentially obliterate a tumor in the dental region.

Arabi and Zaidi^73^ proposed to use deep convolutional neural networks for completion of artifact areas. The authors used co-registered PET, CT, and MR data from 15 patients examined with FDG-PET for staging of head and neck malignancies, to train a network for completion of the MR images. The evaluation was performed using 10 patients with metal-induced artifacts, where using the proposed deep learning architecture improved segmentation accuracy in the area incorrectly segmented as air, decreasing SUVmean bias from −60 *±* 11% (without correction) to −24 *±* 9% (with correction).

A study by Fuin et al.^74^ proposed to use emission data in non-TOF systems to correct the artifacts in the attenuation map. Here, the authors first semiautomatically segmented the artifact region, and then fixated the LACs outside the region during the reconstruction. The method appears to recover not only the shape but also the LAC of the metal implant, and if so, being the only method available to do so. The method was only tested on 11 patients, of which 2 were dental implants, and therefore remains to be tested on a larger patient cohort.

### Whole-body MRAC

Similar to brain MRAC, strategies for metal artifact correction in whole-body MRAC can be classified into different groups that are described in the following.

#### Dedicated MRI sequences

As in the case of brain MRI, dedicated MRI sequences that reduce the size of the signal voids around metal implants such as SEMAC or MAVRIC^1,75–78^ can help to reduce the size of the artifact regions in the MRI-based attenuation images and thus lower the bias introduced in the reconstructed PET images. However, to the best of our knowledge, there are currently no publications that evaluate the performance of those sequences in the context of whole-body MRAC.

#### Inpainting methods

The basic idea of inpainting methods is to fill the signal voids caused by metal implants in the attenuation MRI with the soft-tissue LAC in the corresponding attenuation image. In this way, the bias introduced in the attenuation factors (negative exponential of the forward projected attenuation image) is strongly reduced.

Ladefoged et al.^29^ showed that manual inpainting of the artifact regions with the soft tissue LAC can restore the uptake pattern in non-TOF PET reconstructions in the vicinity of the metal artifact and concluded that this “method can be recommended as a first-line retrospective correction of artifacts resulting from endoprostheses present during WB PET/MRI imaging.” The authors also showed that inpainting with the soft-tissue LAC leads to less bias compared to inpainting with the much higher metal LAC. The latter leads to substantial local overestimations of the reconstructed activity concentration since the signal void in the commonly used attenuation MRI sequences is much bigger than the metal implant itself.

Based on this work, Schramm et al.^42^ presented a method for automatic inpainting of MRAC metal artifact regions with the soft tissue LAC. The proposed method consists of three steps. First, the outer contour of the attenuation MRI is closed based on information obtained from an initial TOF PET reconstruction with a simple segmentation-based attenuation image. This contour closing is needed for lung segmentation in the case of sternal cerclages or for the inpainting of MRI signal voids that are connected to the background such as, *e.g*. caused by knee implants. In the second step, the lung is segmented as low MRI intensity regions at a certain distance to the shoulders. In the final step, all other internal low MRI intensity regions are filled with the soft tissue LAC. The authors demonstrated the stability of the algorithm in 13 patients with sternal cerclages, hip implants and knee implants. A potential drawback of this method is the fact that all internal air cavities outside the lungs are also filled the soft-tissue LAC which leads to local overestimations of the reconstructed activity concentration close to those regions.

#### Atlas-based methods

As discussed before, atlas-based methods for the generation of MRI-based attenuation images, especially those relying on pattern or patch matching, are challenged by bigger regions without any MRI signal.

**Figure 6.**
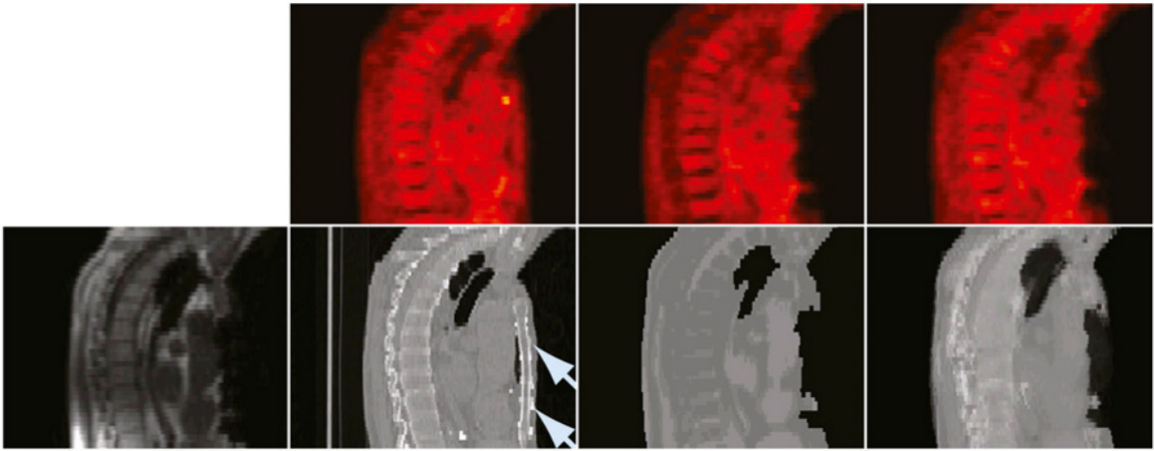
Patient with metal implant in sternum. Bottom row, from left to right: in-phase MR image; CT image, with arrows indicating location of metal implant; pseudo-CT from 5-class MR image segmentation; and AT&PR-based pseudo-CT. Top row: corresponding PET images reconstructed using pseudo-CT images for AC. This research was originally published in JNM. Hofmann et al. “MRI-Based Attenuation Correction for Whole-Body PET/MRI: Quantitative Evaluation of Segmentation- and Atlas-Based Methods ”. *J Nucl Med*. 2011;52:1392–1399. ^©^ SNMMI.^61^

While the atlas registration and pattern recognition method presented by Hofmann et al.^61^ was able to strongly reduce the bias in the reconstructed PET images close to metal artifacts caused by a postoperative clip and a dental implant, it failed to compensate the signal void caused by a sternal wire and thus resulted in substantial negative bias (−40%) in this region as shown in Figure 6.

**Figure 7.**
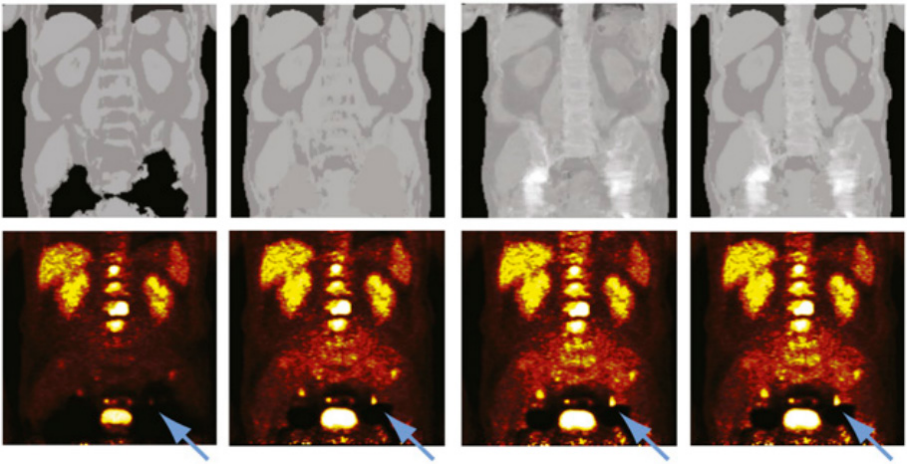
Attenuation maps (top) and PET images (bottom) for sample patient with hip prostheses. The used MRAC methods, from left to right, were SEG1, SEG2, AT&PR, and SEG2wBONE. Metal artifacts are due to bilateral hip replacement. Blue arrows denote lesion affected by presence of adjacent metal artifact when no correction was performed. This research was originally published in JNM. Bezrukov et al. “ MRI-Based Attenuation Correction Methods for Improved PET Quantification in Lesions Within Bone and Susceptibility Artifact Regions” *J Nucl Med*. 2013;54:1–7 ^©^SNMMI.^79^

Based on the work in Hofmann et al.,^61^ Bezrukov et al.^79^ developed two advanced methods for the compensation of MRAC metal artifacts that use a combination of segmentation- and atlas-based prior knowledge. The first method (SEG2) uses an atlas of potential metal artifact regions to fill signal voids caused by metal implants with the soft tissue LAC. The artifact location atlas was derived from 11 patients with metal implants and contained prior information about the location of hip prostheses in the pelvis and about sternal cerclages and portal catheters in the thorax. In the second method (SEG2wBone), information about higher bone attenuation was superimposed on the attenuation images generated with SEG2. Using the developed methods, the bias in nine lesions located close to the metal artifacts regions could be reduced from *−*54 ± 38.4% to *−*15.0 ± 12.2% (SEG2) and to 0.6 ± 11.1% (SEG2wBone). A representative example is shown in Figure 7. As mentioned before, the performance of any atlas-based method strongly depends on the number and quality of data sets available in the atlas.

#### Joint estimation of emission and attenuation

An interesting alternative to all previous methods is the estimation of attenuation properties from the acquired PET data itself. Defrise et al.^80^ could prove that the availability of TOF determines the attenuation sinogram up to a constant. Rezaei et al.^81^ proposed the MLAA estimation algorithm that allows to jointly reconstruct the emission and attenuation image (instead of the attenuation sinogram). The authors could show that with TOF data the cross-talk between emission and attenuation image can be strongly reduced,^82^ provided that the object diameter is bigger than the spatial width of the TOF kernel.

Since MLAA does in general not rely on MRI or CT information, it is not directly affected by metal-induced signal voids in attenuation MR images nor by metal artifacts in attenuation CT images. Note, however, that all joint estimation methods need prior information since the attenuation sinogram is only determined up to a constant. This prior information can be, *e.g*. implemented as an absolute intensity prior for the linear attenuation coefficients of a certain tissue class (*e.g.* soft-tissue) in a given region.

**Figure 8.**
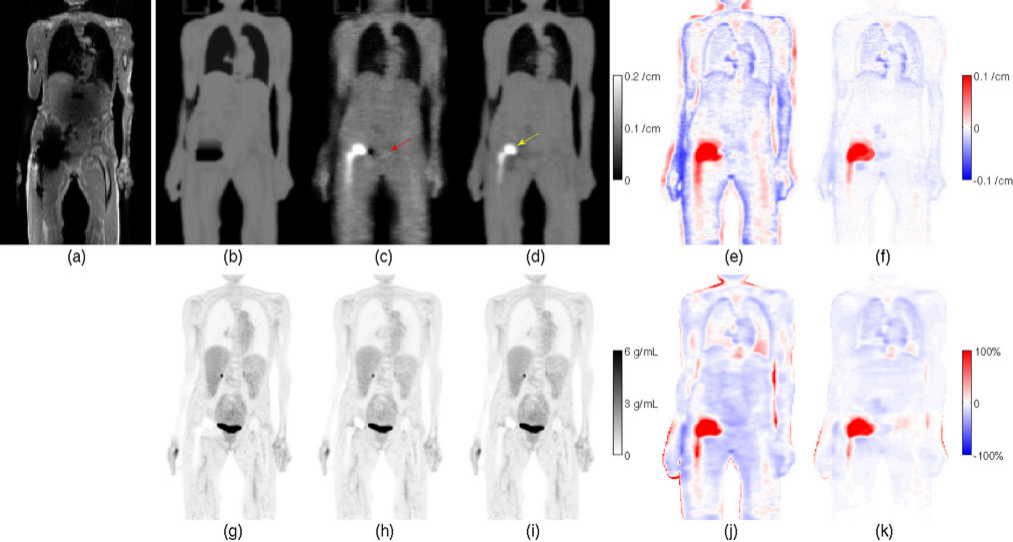
Example coronal slice containing a hip endoprosthesis, for patient 1 with ^18^F-FDG, of (a) in-phase MR image; attenuation maps (b) *µ* ^MR^ based on MR, (c) *µ* ^JEnoMR^ based on JE without MR-based priors, and (d) *µ* ^JE^ based on JE with MR-based priors; differences in the attenuation maps, (e) *µ* ^JEnoMR^ - *µ* ^MR^, and (f) *µ* ^JE^ - *µ* ^MR^; TOF OSEM reconstructed activity images (g) *λ* ^MR^ based on *µ* ^MR^, (h) *λ* ^JEnoMR^ based on *µ* ^JEnoMR^, and (i) *λ* ^JE^ based on *µ* ^JE^; and relative differences in the TOF OSEM reconstructed images, (j) (*λ* ^JEnoMR^ - *λ* ^MR^)/*λ* ^MR^, and (k) (*λ* ^JE^ - *λ* ^MR^)/*λ* ^MR^. The yellow arrow indicates the recovered metallic implant in the JE reconstructed attenuation map in (d), and the red arrow indicates the overestimated attenuation region corresponding to the bladder in *µ* ^JEnoMR^. Reprinted from Ahn et al. “Joint estimation of activity and attenuation for PET using pragmatic MR-based prior : application to clinical TOF PET/MR whole-body data for FDG and non-FDG tracers”, *Phys Med Biol* 2018;63:045006,^83^ https://doi.org/10.1088/1361-6560/aaa8a6. ^©^ Institute of Physics and Engineering in Medicine. Reproduced by permission of IOP Publishing. All rights reserved.

Ahn et al.^58,83^ proposed to combine MLAA with a quadratic intensity prior for the attenuation coefficients based on an initial standard MRI segmentation-based attenuation image. High prior weights were used in regions with known attenuation (regions with high MRI signal) and low prior weights were used in regions with low MRI signal which are potentially lung, air cavities, cortical bone, or metal artifact regions. The authors could show in 12 TOF data sets using 3 different tracers that their proposed method is able to recover the shape and the higher metal attenuation coefficients of a hip implant and pedicle screws in the spine (see Figure 8). Moreover, lower attenuation values in abdominal air cavities could be recovered. The authors could also show that the MRI-based prior substantially reduced residual cross-talk artifacts as observed in the bladder.

**Figure 9.**
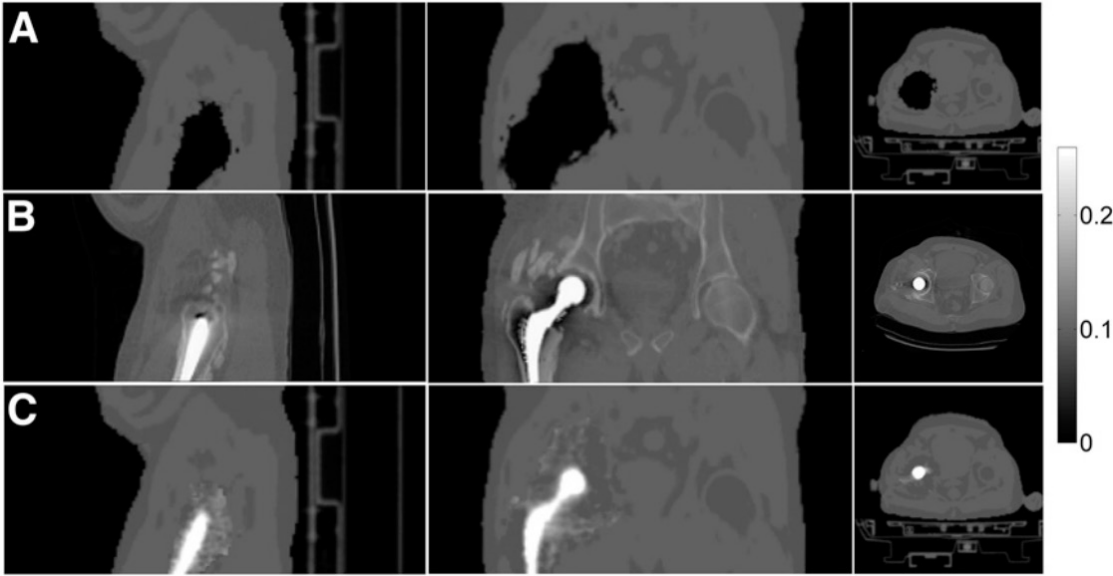
Patient presenting right hip cobalt-chromium alloy endoprosthesis (patient 1). Dixon (A), CT (B), and IPAC (C) *µ*-maps are shown. The three columns show (from left to right) sagittal, coronal, and axial planes. This research was originally published in JNM. Fuin et al. “PET/MR Imaging in the Presence of Metal Implants: Completion of the Attenuation Map from PET Emission Data” *J Nucl Med*. 2017;58:840–845. ^©^ SNMMI.^74^

As mentioned above, MLAA in general suffers from cross-talk artifacts when TOF information is not available. However, Fuin et al.^74^ demonstrated that their algorithm “implant PET-based attenuation map completion” works well with non-TOF data sets from the Siemens mMR (see Figure 9). To circumvent the cross-talk problem, Fuin et al. initialized their joint reconstructions with a MRI segmentation-based attenuation image where the metal artifact regions were manually inpainted with the soft-tissue LAC (similar to^29^). Moreover, in the optimization of the joint cost function using the L-BFGS-B optimizer, the attenuation image was strongly constrained by keeping all attenuation coefficients outside the metal artifact regions fixed. On top of that, the attenuation coefficients within the metal artifact regions were constrained to be within 0.08 and 1 cm*^−^* ^1^. The analysis of 11 patients with 13 metal implants showed that the bias introduced by MRAC metal artifacts could be reduced from 80.1% ± 27.1% to 20.3% ± 23.1% using CTAC as a reference.

## Discussion

All in all, publications on techniques for correction of metal-implant induced artifacts in MRAC are still scarce and no reliable and stable solution has been implemented in any of the currently available PET/MRI systems. As discussed in “The prevalence and impact of metal-implant induced MRAC artifacts on the reconstructed PET image quality and quantification” section, the MRAC metal artifacts can have a severe impact on PET image quantification and lesion detectability in the vicinity of bigger metal implants*—*typically seen in the body*—*depending on the available TOF resolution. While artifacts caused by bigger metal implants are visually easy to detect in the reconstructed PET images, small artifacts as, *e.g*. caused by stents or surgical clips might be missed and might lead to misinterpretations of the reconstructed PET images as shown in Figure 4 depending on the clinical task. It should be once again emphasized that attenuation images should always be inspected for potential (metal) artifacts when reading PET images from PET/MRI acquisitions. Unfortunately, studies evaluating the impact for a given clinical task such as, *e.g*. lesion detection or quantification in bigger patient cohorts with metal implants are still missing.

Given the fact that the available TOF resolution of new PET scanners is steadily improving (nowadays between 300 and 400ps), we are convinced that joint estimation methods for emission and attenuation are probably the most promising candidate to solve the MRAC metal problem in current and upcoming TOF PET/MRI systems. The proof of concept results obtained by Ahn et al.^58,83^ and Fuin et al.^74^ using constrained MLAA to estimate the attenuation of metal implants are very promising for TOF and even non-TOF PET/MRI systems, respectively. To validate and improve the performance and stability of those methods in bigger studies, implementations of these algorithms on the commercially available systems by the vendors would be highly desirable. On top of the ability to estimate attenuation values of metal implants correctly, MLAA type methods also have the advantage that they are by design not susceptible for motion-induced misalignments between emission and attenuation images, *e.g*. caused by respiratory motion. Nevertheless, more research needs to be done to reliably solve the inherent scaling problem of MLAA (*e.g.* by using dedicated MRI-based prior knowledge) and to deal with the increased sensitivity of MLAA with respect to inconsistencies in TOF scatter estimation and scanner calibrations.^59,84^

The future potential of atlas- or deep learning-based methods to predict correct attenuation values from MR images with bigger metal artifacts is at the moment hard to predict. This is due to the fact that (a) the problem is very ill-posed since there is no information in the signal void to distinguish the metal implant itself from surrounding tissue and (b) the success of any atlas or deep learning method strongly depends on the availability of a large number of high quality training data sets which are the moment not available for MRAC metal artifacts. Deep learning generative adversarial networks using unpaired data might be a solution to overcome this limitation. Moreover, the generation of ground truth attenuation images is challenging to the presence CT metal artifacts and the non-trivial scaling CT Hounsfield units to 511 keV attenuation values for metals. Undoubtedly, all classical- or deep learning-based approaches would benefit from reducing or avoiding the signal loss, signal pile-up and geometric distortions around metal implants. Whether this can be achieved with MRI sequences such as MAVRIC or SEMAC within a given amount of available acquisition time, remains to be tested.

From the view point of the current clinical PET/MRI user, there is still no vendor solution to correct for metal MRAC artifacts which means that reliable image reconstruction in the vicinity of bigger metal implants is currently not possible. Until more sophisticated correction methods become available, a manual inpainting method implemented in the clinical systems would be very helpful to correct the major part of the bias caused by MRAC metal artifacts.
